# Syphilis mimicking a metastatic tumour: a case report and review of the literature

**DOI:** 10.1007/s15010-026-02803-0

**Published:** 2026-05-06

**Authors:** Tobias R. Dashi, Sebastian Boeltz, Kristina Schilling, Olaf Conrad, Shahed Shafaei, Eva L. Balbach, Franziska Schmid, Barbara Nuebel, Abbas Agaimy, Jochen Wacker, Giuseppe Valenza, Georg Schett, Thomas Harrer

**Affiliations:** 1 Department of Internal Medicine 3—Rheumatology and Immunology, Friedrich-Alexander-Universität (FAU) Erlangen-Nürnberg and Universitätsklinikum Erlangen, Erlangen, Germany; 2 Department of Internal Medicine 5—Hematology and Oncology, Friedrich-Alexander-Universität (FAU) Erlangen-Nürnberg and Universitätsklinikum Erlangen, Erlangen, Germany; 3 Department of Otolaryngology—Head & Neck Surgery, Friedrich-Alexander-Universität (FAU) Erlangen-Nürnberg and Universitätsklinikum Erlangen, Erlangen, Germany; 4 Department of Nuclear Medicine, Friedrich-Alexander-Universität (FAU) Erlangen-Nürnberg and Universitätsklinikum Erlangen, Erlangen, Germany; 5 Department of Radiology, Friedrich-Alexander-Universität Erlangen-Nürnberg and Universitätsklinikum Erlangen, Erlangen, Germany; 6 Department of Urology and Pediatric Urology, Friedrich-Alexander-Universität (FAU) Erlangen-Nürnberg and Universitätsklinikum Erlangen, Erlangen, Germany; 7 Institute of Pathology, Friedrich-Alexander-Universität (FAU) Erlangen-Nürnberg and Universitätsklinikum Erlangen, Erlangen, Germany; 8 Mikrobiologisches Institut—Klinische Mikrobiologie, Immunologie und Hygiene, Friedrich-Alexander-Universität (FAU) Erlangen-Nürnberg and Universitätsklinikum Erlangen, Erlangen, Germany; 9 Infectious Diseases Section, Department of Internal Medicine 3—Rheumatology and Immunology, Friedrich-Alexander-Universität (FAU) Erlangen- Nürnberg and Universitätsklinikum Erlangen, Erlangen, Germany

**Keywords:** Tertiary syphilis, Treponema pallidum, Pseudotumour, Infectious diseases

## Abstract

**Purpose:**

Syphilis is an infectious disease with a wide range of clinical manifestations. Diagnosis is often challenging as the symptoms of syphilis can mimic malignant or autoimmune diseases. Here, we report on a patient with tertiary syphilis mimicking a metastatic testicular tumour.

**Methods:**

Syphilis was diagnosed by serological testing and immunohistochemistry of tissue biopsies using specific antibodies. Additionally, a comprehensive literature review was conducted using a PubMed database search to identify previously reported cases and relevant studies.

**Results:**

A 57-year-old male patient developed fever, night sweats and weight loss for three months after he had been treated with glucocorticoids for lumbar disc herniation. At first presentation, physical examination was unremarkable except for cervical lymphadenopathy. FDG-PET revealed hepatic and testicular lesions suspicious for malignancy. Biopsy of the hepatic lesion was inconclusive and showed features of pseudotumourous inflammation. The orchiectomy specimen demonstrated a large inflammatory pseudotumour composed of mixed lymphoplasmacytic and granulocytic infiltrates, with no evidence of neoplasia. Serological testing yielded elevated titers of Treponema pallidum antibodies and immunohistochemistry detected high numbers of spirochaetes in the biopsies. Antibiotic treatment with ceftriaxone and benzylpenicillin led to relief of symptoms and a decline of antibody titers. Literature review identified several cases of syphilis masquerading as malignant disease, whereas a potential reactivation of syphilis under immunosuppressive therapy remains rare.

**Conclusion:**

This case highlights tertiary syphilis as a rare but important differential diagnosis of pseudotumorous lesions mimicking metastatic malignancy, particularly in the presence of atypical histopathology. Immunosuppression may have contributed to clinical manifestation.

## Introduction

Syphilis is a sexually transmitted chronic disease caused by Treponema pallidum. Symptoms differ depending on disease stage. Following infection, painless genital, anal, or oral ulcers (chancres) typically emerge at the site of inoculation and may be accompanied by regional swelling of lymph nodes. These lesions may resolve spontaneously, but sometimes, local proliferation of the spirochaetes can also induce tumorous mucocutaneous manifestations. Dissemination of the bacteria can lead to a variety of symptoms such as maculopapular rash, condylomata lata, alopecia, vasculitis, meningitis, cranial neuritis, uveitis, hepatitis and nephritis. In untreated individuals, late manifestations may develop after years of chronic infection and can result in cardiovascular complications such as aortitis, aortic aneurysm and aortic valve disorder. Late neurosyphilis may lead to chronic meningitis, cerebral vasculitis, tabes dorsalis and general paresis, which may be associated with progressive dementia and personality disorders [1]. A hallmark of tertiary syphilis is the formation of gummata, representing destructive granulomatous lesions that may occur in virtually any organ, most commonly affecting the skin, bone, and visceral tissues [2]. Importantly, tertiary syphilis may present as pseudotumorous lesions closely mimicking malignant disease, posing a significant diagnostic challenge. Due to this broad and often non-specific clinical spectrum, syphilis remains a diagnostic pitfall. Here, we report a case of tertiary syphilis with testicular involvement presenting as a metastasis-mimicking lesion, highlighting the importance of including syphilis in the differential diagnosis of such presentations.

## Methods

Imaging was performed using 18-fluoro-2-deoxyglucose positron emission tomography. Treponema pallidum-specific antibodies were measured using the following methods: Treponema pallidum screening test: Treponema pallidum Screen-EIA (Euroimmun, Lübeck, Germany). Treponema pallidum hemagglutination test: TPHA-Test (DIALAB GmbH, Vienna, Austria). Treponema pallidum IgM-FTA-absorption test: Mastafluor FTA-Abs. Kit, Treponema pallidum (MAST Group, Reinfeld, Germany). Cardiolipin assays: Rapid Plasma Reagin-Test (Bio-Rad, Marnes-la Coquette, France). Tissue biopsies were analysed for spirochaetal infection using a Treponema pallidum-specific polyclonal antibody (Biocare Medical, Pacheco, USA), applied at a 1:100 dilution and incubated at 37 °C for 32 min. Informed consent for publication of the case was obtained. A systematic literature review was conducted in November 2025 using PubMed to identify reports of tertiary syphilis presenting as pseudotumourous lesions using the following keywords and operators: (syphilis [MeSH Major Topic] OR Treponema pallidum [MeSH Major Topic]) AND ((pseudotumor OR malignancy) AND mimicking) AND (case reports [Publication Type]). Inclusion criteria comprised published case reports describing syphilis presenting as pseudotumorous lesions or mimicking malignancy, with available clinical or histopathological data; studies were excluded if key variables (publication details, age, clinical or histological findings, suspected malignancy) were missing, except for sex. Two authors independently screened records with consensus resolution. Data extracted included publication details, age, sex, clinical and histological findings, and suspected malignancy. Of 68 records, 15 were excluded. The review was conducted in accordance with PRISMA principles.

## Case report

A 57-year-old man developed cervical lymphadenopathy and constitutional symptoms (night sweats, weight loss of 13 kg). After seven months of persistent symptoms, he was referred to our hospital for further evaluation. Laboratory analyses revealed a slight increase in C-reactive protein to 29.5 mg/l (normal range < 5), a slight decrease of hemoglobin to 12.3 g/dl (normal range 13.5–17) and a polyclonal elevation of gamma-globulins to 22% in the protein electrophoresis (normal range 11–19%), with increase in IgG3 (172.9 mg/dl, normal range 24–125) and IgG1 (1062 mg/dl, normal range 280–800). Except for a slightly elevated γ-glutamyltransferase with 65 U/l (normal range < 60) and an elevation of alkaline phosphatase to 215 U/l (normal range 40–130), further blood chemistry analyses including creatinine, ALT, AST, and LDH were all within the normal range (Table 1). Serological testing found no evidence of a variety of infections, such as HIV-1, hepatitis B, hepatitis C, human herpesvirus 8 (HHV-8), bartonellosis, brucellosis, tularemia, entamoebiasis, and toxoplasmosis. A tuberculosis interferon-gamma release assay (IGRA) was negative. EBV and CMV serology indicated past infections. Ultrasound confirmed cervical lymphadenopathy and additionally revealed liver lesions suspicious for malignancy.

**Fig. 1:**
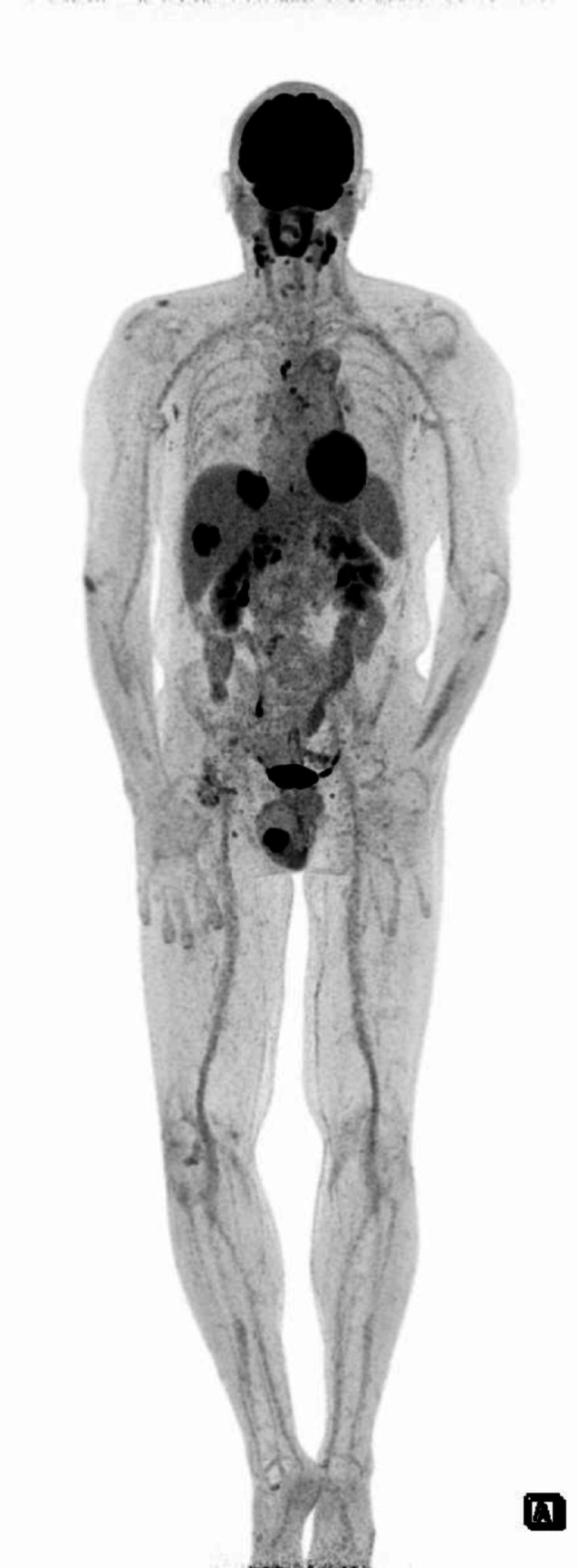
18 F-FDG PET CT demonstrating hypermetabolic lesions in the liver, lymph nodes, and testis

**Fig. 2:**
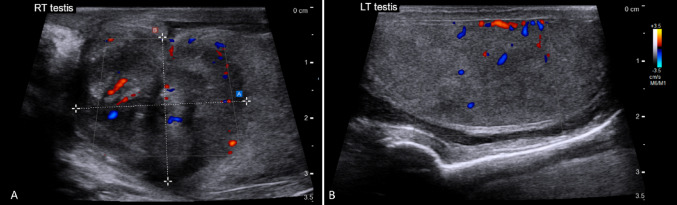
Scrotal ultrasound revealing an inhomogeneous testicular mass with irregular margins and increased perfusion (**A**), with a normal-appearing contralateral testis shown for comparison (**B**)

**Fig. 3:**
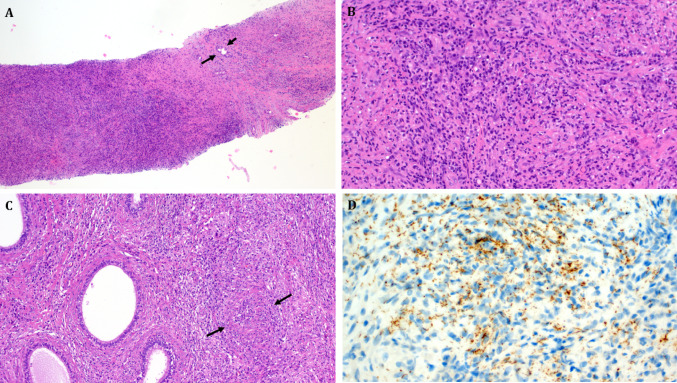
Liver and testis histopathology with spirochaete staining. **A** A core needle biopsy of the liver shows confluent/tumefactive mixed mononuclear inflammatory infiltrate with few bile duct remnants (arrows). **B** Higher magnification of A. **C** The testis shows similar tumefactive inflammation with scattered epididymal ducts in the background, note focal obliterative angiitis (arrows). **D** Spirochaete-specific immunohistochemistry highlighted extensive spiral bacteria (consistent with *Treponema pallidum*) in the liver biopsy (image) and in the testicular lesion (not shown)

A subsequent fluorodeoxyglucose positron emission tomography (18 F-FDG PET) CT scan showed metabolically active abdominal and perihilar lymphadenopathy, hepatic lesions, and increased testicular tracer uptake (Fig. 1). Scrotal ultrasonography demonstrated an irregularly delineated, inhomogeneous testicular mass with increased vascularity (Fig. 2). A liver biopsy was performed and histopathological analysis showed necrosis of liver tissue with mixed lymphoplasmacytic and granulocytic inflammatory cell infiltrates (Fig. 3a and b). Staining for mycobacteria, CMV and IgG4^+^ plasma cells was negative. Persistent testicular swelling, pain, and suspicious ultrasound findings prompted a unilateral orchiectomy after multidisciplinary tumour board review. Histology of the testicular tissue revealed marked chronic lymphocytic inflammation with fibrosis and necrosis, consistent with an idiopathic inflammatory pseudotumour (Fig. 3c).

After repeated questioning, the patient reported a history of lumbar disc herniation 10 months before admission. He was treated with two intramuscular injections of glucocorticoids and three months later again with two courses of oral prednisolone over seven days without improvement of back pain. Thereafter, he developed profuse night sweats, weight loss, cervical lymphadenopathy and an aggravation of his long-standing headaches. As we realized that his symptoms began shortly after the initiation of glucocorticoid therapy, further evaluation for infectious diseases was performed, including screening for syphilis. Syphilis serology showed a strong positive reaction with high antibody titers of 1:81920 in the TPHA assay and 1:256 in the cardiolipin assay. Immunohistochemical staining of previously obtained liver and testicular tissues demonstrated abundant spirochaetes in the liver and sparse organisms in the testicular tissue, consistent with tertiary syphilis (Fig. 3d).

**Table 1:** Routine laboratory blood test results at initial evaluation

Hemoglobin	12.3 g/dl	Sodium	140 mmol/l	Gamma-globulin	22%
Hematocrit	38.2%	Creatinine	0.84 mg/dl	Alkaline phosphatase	215 U/l
Leukocytes	8.93 × 10^3^/µl	C-reactive protein	29.5 mg/l	γ-GT	65 U/l
Platelets	304 × 10^3^/µl	AST / GOT	28 U/l	IgG1	1062 mg/dl
Neutrophils	5.93 × 10^3^/µl	ALT / GPT	25 U/l	IgG3	172.9 mg/dl
Eosinophils	0.09 × 10^3^/µl	LDH	175 U/l	CSF leucocytes	1 /µl
Basophils	0.02 × 10^3^/µl	T cells (CD3+)	1882 cells/µl	CSF protein	321 mg/l
Lymphocytes	2.19 × 10^3^/µl	T helper cells (CD3+ / CD4+)	1060 cells/µl	CSF glucose	67 mg/dl
Monocytes	0.70 × 10^3^/µl	B lymphocytes (CD19+)	281 cells/µl	CSF lactate	1.8 mmol/l
Potassium	4.6 mmol/l	Activated partial thromboplastin time	43.3 s	ITPA index	0.9

CSF, cerebrospinal fluid; ITPA, intrathecal produced T. pallidum antibodies.

The patient initially received empiric intravenous ceftriaxone (2 g/day) for two days, given its efficacy against neurosyphilis and the initial clinical suspicion while the diagnosis was still under evaluation. Subsequent assessment showed no evidence of neurosyphilis: Ophthalmoscopy showed no signs of neuro-ocular involvement, and cerebrospinal fluid analysis revealed neither pleocytosis nor intrathecal antibody synthesis against Treponema pallidum according to the Reiber scheme; the intrathecal produced T. pallidum antibodies (ITPA) index of 0.9 was within the normal range, arguing against intrathecal antibody production. This is consistent with recent multicentre data suggesting a cutoff of 1.7 for the ITPA index [3]. Therefore, according to current guidelines for the treatment of tertiary syphilis without neuro-ocular involvement, treatment was switched to intramuscular depot benzathine benzylpenicillin (2.4 million IU weekly for three weeks), resulting in relief of symptoms and a decline in inflammation markers. The patient reported multiple heterosexual contacts before his marriage 20 years ago, but he denied extramarital sexual contacts. He did not remember any skin symptoms of primary stage syphilis. He was never treated for syphilis, but he presumably had received perioperative antibiotics for surgery of the shoulders and hip 12, 13, and 18 years ago, and one-course of an oral antibiotic for a traumatic finger infection 21 years ago.

## Literature review

A review of the literature on syphilis infections mimicking malignant disease identified 53 publications, comprising 52 single-patient case reports and one case series involving three patients, revealing the following patterns: The majority of affected individuals were male (approximately 3:1), with a mean age of 42.7 years (IQR 32–52). Most reported cases originated from Asia (over 50%), followed by Europe (approximately one quarter). The most common presenting symptoms were those related to the central nervous system, such as seizures and headaches, accounting for about 30% of cases, followed by perianal lesions (13%) (Table 2). However, it must be considered that CNS lesions are more likely to be published than less severe manifestations, such as perianal lesions or localized ulcerations, which may lead to reporting bias [4–56].

In most reported cases, a biopsy was performed before serological testing for syphilis. This approach carries a substantial and often avoidable risk of surgical complications, particularly in CNS presentations, and could potentially be circumvented through earlier serological evaluation.

**Table 2:** Demographic characteristics and clinical findings

Origin of Publications [*n*_1_] (%)		Total [53] (100%)
Asia		27 (50.9%)
Europe		15 (28.3%)
North America		6 (11.3%)
South America		5 (9.4%)
Africa		0 (0%)
Sex [*n*_2_] (%)		[55] (100%)
Male		39 (70.9%)
Female		13 (23.6%)
n/a		3 (5.5%)
Mean age [years] (Interquartile range)		42.7 (32–52)

Physical findings [*n*_1_] (%)	Suspected malignancy	Total [53] (100%)
B symptoms, disseminated lymphadenopathy	Lymphoma	3 (5.7%)
Epigastric pain, anorexia, jaundice	Gastrointestinal carcinoma	5 (9.4%)
Oral ulcers, tongue nodules, cervical masses	Oropharyngeal cancer	6 (11.3%)
Perianal lesions	HPV-tumour/rectal cancer	7 (13.2%)
Pleuritic pain, osteolytic lesions, vaginal bleeding	Lung carcinoma, bone metastasis	6 (11.3%)
Seizures, headaches, visual/hearing symptoms	Brain tumour/abscess	16 (30.2%)
Skin rash, infiltrated plaques, nodular lesions	Cutaneous lymphoma	7 (13.2%)
Testicular mass	Testicular cancer	3 (5.7%)

Origin of publications, sex distribution, mean age, clinical findings and suspected malignancy of reported cases of syphilis mimicking malignancy; one publication reported three patients of unknown sex, which explains the differing denominators used for sex distribution; n/a, not available; n_1_: number of publications; n_2_: number of patients

## Discussion

Tertiary syphilis can present decades after initial infection and may closely mimic metastatic malignancy, particularly when lesions are pseudotumourous. In this case, diagnosis and treatment were delayed due to atypical presentation and the prolonged latency of infection. The patient denied any history of syphilis or recent extramarital sexual activity, reporting only multiple sexual contacts more than 20 years ago. Short-term antibiotic therapies, including perioperative prophylaxis, may have suppressed infection without eradicating Treponema pallidum. Histologically, the lesions differed from classic gummata, which typically show central necrosis surrounded by lymphocytes and plasma cells with peripheral fibrosis and epithelioid cells forming a structured granulomatous pattern [2]. Instead, both liver and testis lesions demonstrated dense, mixed lymphoplasmacytic and granulocytic infiltrates, necrosis, fibrosis, and prominent vascular changes, without organized granulomatous architecture. Although classic gummatous features were absent, the overall histopathological findings in the hepatic and scrotal lesions are most consistent with tertiary syphilis. This atypical histopathology underscores the diagnostic challenge of syphilitic pseudotumorous lesions and highlights the need to consider syphilis in the differential diagnosis of inflammatory masses mimicking malignancy, particularly given that earlier serological testing might have avoided orchiectomy in this patient. However, given the imaging findings and concern for malignancy, surgical management was clinically justified at the time. Reactivation of syphilis has been primarily documented in immunosuppressed patients presenting with secondary syphilis symptoms [57–59]. In the present case, prolonged prednisone therapy may have contributed to immune modulation and facilitated reactivation of latent Treponema pallidum infection. As adaptive immunity against Treponema pallidum relies on T cells, prolonged glucocorticoid or immunosuppressant exposure can facilitate reactivation. Macrophages and dendritic cells present treponemal antigens to naïve B and T cells, which differentiate into T-helper cells secreting interferon-gamma, recruiting macrophages to clear bacteria. T. pallidum can evade immune clearance by infiltrating immune-privileged sites such as the CNS and through antigenic variation via the TprK gene, altering surface antigens to escape antibodies [60]. These mechanisms may have contributed to the clinical manifestation in our patient despite elevated IgG and TPPA antibody levels.

This case demonstrates tertiary syphilis after a latency exceeding 20 years, with brief immunosuppression and no comorbidities. It highlights the importance of screening for latent syphilis prior to immunosuppressive therapy, especially given the increasing incidence of syphilis in Germany of approximately five cases per 100,000 inhabitants [61], while atypical histopathological findings highlight the diagnostic complexity.

## Data Availability

No datasets were generated or analysed during the current study.
